# P-1741. The CArbapenem Sparing Antimicrobial Stewardship (CASAS) Project: an innovative approach to reduce carbapenem consumption in low-users hospital settings

**DOI:** 10.1093/ofid/ofae631.1904

**Published:** 2025-01-29

**Authors:** Marco Bongiovanni, Beatrice Barda, Caroline Di Benedetto, Luigia Elzi, Matteo Mombelli, Niccolò Ramponi, Enos Bernasconi

**Affiliations:** Ente Ospedaliero Cantonale, Lugano, Ticino, Switzerland; Ente Ospedaliero Cantonale, Lugano, Ticino, Switzerland; Ente Ospedaliero Cantonale, Lugano, Ticino, Switzerland; Ente Ospedaliero Cantonale, Lugano, Ticino, Switzerland; Ente Ospedaliero Cantonale, Lugano, Ticino, Switzerland; Ente Ospedaliero Cantonale, Lugano, Ticino, Switzerland; Servizio Malattie infettive, Ente Ospedaliero Cantonale, Ospedale, Lugano, Ticino, Switzerland

## Abstract

**Background:**

One of the main objective of the Antimicrobial Stewardship programmes (ASp) is the optimization of antibiotic use, reducing the use of broad spectrum antibiotics when unnecessary. In this context, the close monitoring of carbapenem consumption is mandatory.

**Figure:**
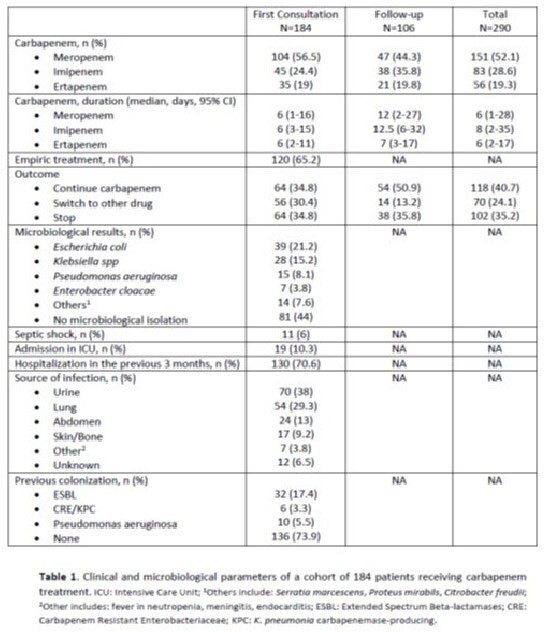

**Methods:**

The CASAS Project started in January 2024 in 4 public Hospitals (for a total of approximately 1000 beds) in Southern Switzerland, a region with a low prevalence of multi-drug-resistant bacteria (MDR) and with a low use of carbapenem treatments (CAR-T). An electronic alert was sent to the Infectious Diseases (ID) Team after 96 hours of continuous CAR-T; at that point, an ID specialist review was performed in order to confirm or not the CAR-T. For patients who continued CAR-T ID consultation was repeated every 72 hours until stopping. Parameters included in the consultation were: empiric or targeted treatment, type of carbapenem (meropenem, imipenem or ertapenem) and duration, outcome (confirm CAR-T, stop or switch to other drug), microbiological results, septic shock, admission to ICU, hospitalization in the previous 3 months, source of infection and known colonization with MDR. A descriptive analysis of these parameters was performed and finally we compare the Defined Daily Dose (DDD) of each carbapenem in the first trimester of 2023 and 2024.

**Results:**

A total of 290 consultations were performed between Jan 1 and Mar 31, 2024 (184 first and 106 follow-up visits). Meropenem was the most used CAR-T (n=151) for a median duration of 13 days (4-14). Table 1 summarizes the other parameters considered in the analysis. An overall reduction of DDD for carbapenems was observed (overall DDD: 2023: 2372.6; 2024: 2026.5, -14.6%). When splitting the results for the single carbapenem, the main reduction was observed for meropenem (DDD 2023: 1563.7; 2024: 1236.4, -20.9%); the reduction for imipenem and ertapenem was -2.3% and -2.4%, respectively.

**Conclusion:**

ASp should include a close monitoring of wide-spectrum antibiotic treatment, including carbapenems. In our setting, the CASAS Project was associated with a rapid reduction in CAR-T also in low-users hospitals. The long-term implementation of similar projects could have a beneficial impact on the prevalence of multi-drug resistance infections.

**Disclosures:**

**All Authors**: No reported disclosures

